# Methylprednisolone Pulses in Hospitalized COVID-19 Patients Without Respiratory Failure: A Randomized Controlled Trial

**DOI:** 10.3389/fmed.2022.807981

**Published:** 2022-02-28

**Authors:** Iñigo Les, Jose Loureiro-Amigo, Ferran Capdevila, Isabel Oriol, Iñaki Elejalde, Judit Aranda-Lobo, Joao Modesto, Elena Güell-Farré, Ruth García, Anna Murgadella-Sancho, Javier Anniccherico, Miguel Martín-Fernández, José Javier Lorza, Joan-Pol Monteys-Montblanch, Julián Librero, Sara Pintado-Lalueza, Marina Delgado, Berta Gracia-García, Julio Sánchez-Álvarez, Melani Pestaña-Fernández, Patricia Fanlo, Gisela Funalleras-Puig, Maite Sarobe, Eduardo Mediavilla, Carlos Ibero

**Affiliations:** ^1^Internal Medicine Department, Navarra Hospital Complex, Pamplona, Spain; ^2^Autoimmune Diseases Unit, Internal Medicine Department, Navarra Hospital Complex, Pamplona, Spain; ^3^Internal Medicine Department, Moisès Broggi Hospital, Consorci Sanitari Integral, Sant Joan Despí, Barcelona, Spain; ^4^Hospital Pharmacy Department, Navarra Hospital Complex, Public University of Navarra, Healthcare Research Institute of Navarra, Pamplona, Spain; ^5^Infectious Diseases Unit, Internal Medicine Department, Moisès Broggi Hospital, Consorci Sanitari Integral, Sant Joan Despí, Barcelona, Spain; ^6^Clinical Trials Platform, Navarrabiomed, Navarra Hospital Complex, Public University of Navarra, Healthcare Research Institute of Navarra, Pamplona, Spain; ^7^Hospital Pharmacy Department, Moisès Broggi Hospital, Consorci Sanitari Integral, Sant Joan Despí, Barcelona, Spain; ^8^Pneumology Department, Navarra Hospital Complex, Pamplona, Spain; ^9^Navarrabiomed, Navarra Hospital Complex, Public University of Navarra, Healthcare Research Institute of Navarra, Pamplona, Spain; ^10^Medical Oncology Department, Navarra Hospital Complex, Pamplona, Spain; ^11^Emergency Department, Navarra Hospital Complex, Pamplona, Spain; ^12^Infectious Diseases Unit, Navarra Hospital Complex, Pamplona, Spain

**Keywords:** corticosteroids, methylprednisolone, COVID-19, SARS-CoV-2, hospitalized patients, randomized controlled trial

## Abstract

**Background:**

Corticosteroids are the cornerstone of the treatment of patients with COVID-19 admitted to hospital. However, whether corticosteroids can prevent respiratory worsening in hospitalized COVID-19 patients without oxygen requirements is currently unknown.

**Aims:**

To assess the efficacy of methylprednisolone pulses (MPP) in hospitalized COVID-19 patients with increased levels of inflammatory markers not requiring oxygen at baseline.

**Methods:**

Multicenter, parallel, randomized, double-blind, placebo-controlled trial conducted in Spain. Patients admitted for confirmed SARS-CoV-2 pneumonia with raised inflammatory markers (C-reactive protein >60 mg/L, interleukin-6 >40 pg/ml, or ferritin >1,000 μg/L) but without respiratory failure after the first week of symptom onset were randomized to receive a 3-day course of intravenous MPP (120 mg/day) or placebo. The primary outcome was treatment failure at 14 days, a composite variable including mortality, the need for ICU admission or mechanical ventilation, and clinical worsening, this last parameter defined as a PaO_2_/FiO_2_ ratio below 300; or a 15% decrease in the PaO_2_ from baseline, together with an increase in inflammatory markers or radiological progression. If clinical worsening occurred, patients received tocilizumab and unmasked corticosteroids. The secondary outcomes were 28-day mortality, adverse events, need for ICU admission or high-flow oxygen, length of hospital stay, SARS-CoV-2 clearance, and changes in laboratory parameters.

**Results:**

A total of 72 patients were randomized and 71 patients were analyzed (34 in the MPP group and 37 in the placebo group). Twenty patients presented with treatment failure (29.4 in the MPP group vs. 27.0% in the placebo group, *p* = 0.82), with no differences regarding the time to treatment failure between groups. There were no cases of death or mechanical ventilation requirements at 14 days post-randomization. The secondary outcomes were similar in MPP and placebo groups.

**Conclusions:**

A 3-day course of MPP after the first week of disease onset did not prevent respiratory deterioration in hospitalized COVID-19 patients with an inflammatory phenotype who did not require oxygen.

## Introduction

The coronavirus disease 2019 (COVID-19), caused by the severe acute respiratory syndrome coronavirus 2 (SARS-CoV-2), became a pandemic in March 2020 (1). Accepting disparities among different health systems and viral variants in terms of hospitalization and mortality rates, in our setting, around 20% of patients with COVID-19 need hospital admission and the most severe cases (around 5%) develop acute respiratory distress syndrome (ARDS) requiring admission to an intensive care unit (ICU) (2, 3). Overall mortality by COVID-19 among hospitalized patients ranges between 21 and 26%, with advanced age, male sex, and the existence of comorbidities being the strongest predictors of in-hospital mortality (4–6).

It has been hypothesized that the clinical course of COVID-19 comprises three stages: an initial viremic phase characterized by a viral infection in the respiratory tract; a second pulmonary phase characterized by viral pneumonia; and a third inflammatory phase, starting 2 weeks after the initial infection, characterized by severe pneumonitis eventually leading to the onset of ARDS (7). Thus, many immunomodulatory therapies aiming at mitigating the inflammatory response have been evaluated for the treatment of COVID-19. Corticosteroids block the efflux of neutrophils and monocytes toward inflammatory sites and inhibit numerous pro-inflammatory cytokines (8, 9). So, ever since Wu et al. suggested that methylprednisolone (MP) might be beneficial in COVID-19 patients with ARDS, many studies on the use of corticosteroids for COVID-19 have been reported (10). The Randomized Evaluation of COVID-19 Therapy (RECOVERY) trial was the first randomized clinical trial (RCT) to report that the use of dexamethasone reduced 28-day mortality among patients requiring oxygen or mechanical ventilation (MV), but not among those not receiving supplementary oxygen (11). Since its publication, dexamethasone at a dosage of 6 mg/day for ten days or until discharge consequently became the standard of care for hospitalized COVID-19 patients requiring respiratory support. However, the optimal timing and severity threshold for the initiation of corticosteroids and the dosage to be used are still unclear. Some authors have advocated for the use of pulsed corticosteroids, instead of low to medium doses, to achieve a prompt and strong anti-inflammatory effect while minimizing side effects (12, 13). We hypothezided that a 3-day course of MPP administered after 1 week of symptom onset would interrupt the disease's progression to respiratory failure in COVID-19 patients with an inflammatory phenotype before they need oxygen therapy.

The aim of this clinical trial was to evaluate the impact of methylprednisolone pulses (MPP) on the clinical course of patients with SARS-CoV-2 pneumonia and increased levels of inflammatory markers who had no oxygen requirements.

## Materials and Methods

### Study Design

The CORTIVID trial was a randomized, double-blind, placebo-controlled, parallel design trial (14). The trial was conducted in accordance with the Good Clinical Practice guidelines of the International Council for Harmonization (ICH) E6, the principles of the Declaration of Helsinki, and local regulations. The protocol was reviewed and approved by the institutional review board, and the Spanish Agency of Medicines and Medical Devices (AEMPS). The study was registered in ClinicalTrials.gov (NCT04438980).

### Participants

The trial was conducted in two Spanish sites: the Navarra Hospital Complex (Pamplona, Navarra) and the Moisès Broggi Hospital (Sant Joan Despí, Barcelona). Patients were identified, screened and invited to participate in the trial at the Emergency Department or the hospitalization ward. During their hospital stay, the patients were followed-up in accordance with local protocols. More details on the patients' standard of care procedures are available in the Supplementary Material section corresponding to the study protocol.

Patients were eligible if they were ≥18 years old; had been admitted with SARS-CoV-2 pneumonia confirmed by a positive reverse-transcription polymerase chain reaction (RT-PCR) of nasopharyngeal swab, defined as a cycle threshold (Ct) ≤ 35; presented with symptoms compatible with COVID-19 for at least 7 days; and had at least one of the following: C-reactive protein (CRP) >60 mg/L, interleukin (IL)-6 > 40 pg/ml, or ferritin >1,000 μg/L. Patients were excluded from the trial if they had allergy to or contraindication for corticosteroids; an indication other than COVID-19 for treatment with corticosteroids or immunosuppressants; and respiratory failure, defined as a peripheral oxygen saturation (SpO_2_) below 90% in ambient air or a ratio between the arterial partial pressure of oxygen (PaO_2_) and the fraction of inspired oxygen (PaO_2_/FiO_2_) below 300. As a result of the use of dexamethasone as the standard of care in June 2020, patients with a SpO_2_ < 94% in ambient air were excluded from the study as of July 2020. Additional exclusion criteria were decompensated diabetes mellitus; uncontrolled hypertension; active cancer; conservative or palliative management; a suspected or confirmed active infection other than SARS-CoV-2; pregnancy or breastfeeding; or participation in another clinical trial. Written informed consent was obtained from all patients. Despite the absence of oxygen requirements at baseline, the hospital admission was justified by the need for close monitoring in patients with pneumonia and raised inflammatory markers. Hospital admission was decided before patients' randomization, at the discretion of the attending physician. During the study period, the criteria for admission did not change in either of the two enrolling hospitals.

Variables regarding the patients' demographic characteristics, medical history, comorbidities, medications administered during the study, time elapsed from symptom onset, SpO_2_ and SpO_2_/FiO_2_ at randomization, and daily post-randomization, chest X-ray findings, and baseline and evolutive laboratory parameters were collected.

### Randomization and Interventions

Randomization was generated by an independent methodologist with the randomize R package of the R software, using two blocks sizes (sized four and six, respectively), with random permutation. The block randomization was stratified by age into two groups (<75 years and ≥75 years). The Pharmacy Department of each institution held the random allocation sequence. Once a patient was enrolled, a prescription was sent to an un-blinded pharmacist who prepared the study drug, and dispensed it in opaque plastic bags containing a total volume of 100 ml and labeled for each participant. Neither the medical team nor the nurses administering the treatment were aware of the content of the bags. Similarly, the physicians who assessed the primary and secondary outcomes, including adverse events, were blinded to treatment assignment.

Eligible patients were randomly assigned at a 1:1 ratio to receive 120 mg of pulsed MP intravenously (experimental group) or placebo (100 ml of 0.9% sodium chloride) intravenously (control group) once daily for 3 consecutive days. Standard of care according to local practice was provided to both groups and could include the use of antiviral drugs (remdesivir, hydroxychloroquine, or lopinavir/ritonavir). On 20 July 2020, the trial's scientific committee introduced a change to remove the use of hydroxychloroquine from the study protocol. This amendment was later approved by the AEMPS on 29 August 2020. The use of corticosteroids outside the protocol was considered a protocol deviation. If the criteria for clinical worsening were met at any point, the patients received intravenous tocilizumab at a dose of 400 mg (<75 kg) or 600 mg (≥75 kg), in addition to unmasked corticosteroids at the discretion of the treating physician. If the patient's clinical signs or symptoms did not improve, a second infusion of tocilizumab could be administered 24 h after the first dose.

The primary analysis was performed at day 14 post-randomization, and the final trial visit took place 28 days after the randomization. Patients who were discharged from the hospital before this date were contacted by telephone.

### Outcomes

The primary outcome was treatment failure, defined as death, ICU admission, initiation of MV, or clinical worsening up to 14 days post-randomization. Clinical worsening was considered when at least one of the following two conditions was met: (1) SpO_2_ in ambient air below 90%, PaO_2_ in ambient air below 60 mmHg, or PaO_2_/FiO_2_ ratio below 300 mmHg; or (2) at least 15% decrease in the PaO_2_ from baseline, together with an increase in the levels of any inflammatory marker (CRP, IL-6, or ferritin) or radiological progression. On 29 June 2020, the trial's scientific committee introduced this second condition as amendment to the protocol, which was approved by the AEMPS on 1 July 2020; at that point, two patients had been included in the study. Key secondary efficacy outcomes were 28-day mortality, the proportion of patients admitted to the ICU or requiring high-flow oxygen therapy within 28 days of the randomization, and the length of hospital stay. Other secondary outcomes included the rate of predefined adverse events (including bacterial, fungal, or opportunistic infections), changes in the levels of inflammatory parameters (CRP, IL-6, ferritin, and D-dimer) within 14 days of the randomization, and viral clearance defined as a Ct >35 in a nasopharyngeal swab RT-PCR at day 7 post-randomization.

### Sample Size and Statistical Analysis

It was estimated that 30 patients per group would be needed to detect an absolute difference of 25% as significant (two-tailed type I error of 0.05 and type II error of 0.2) in the primary outcome: 35 in the control group and 10% in the experimental group. Assuming a loss to follow-up of 20%, we calculated that 72 patients were needed.

Quantitative variables were reported as mean ± standard deviation or median (interquartile range) and compared between groups using Student's *t*-test for unpaired data or Mann-Whitney's *U* test as appropriate. Categorical variables were expressed as frequencies (percentages) and compared between groups using Pearson's χ^2^ or Fisher's exact tests as appropriate.

First, we performed a descriptive bivariate analysis to assess whether randomization achieved a balance between both groups. Then, we compared the primary and secondary outcomes by a modified intention-to-treat analysis in all patients who underwent randomization and received at least one dose of MP or placebo. A multivariate analysis was performed using logistic regression to assess the risk of treatment failure, estimating the effect of the intervention as an odds ratio (OR). Patient survival was assessed using a Kaplan-Meier analysis and compared between groups using the log-rank Mantel-Cox test. Two-sided hypothesis tests were performed, and the significance level was set at 5%. The statistical analysis was conducted using IBM SPSS Statistics software version 27.0 (Chicago, IL), Stata software version 15.1 (Stata Corp., College Station, TX, USA), and R Statistical Software (Foundation for Statistical Computing, Vienna, Austria).

## Results

### Participant Flow

**Figure 1:**
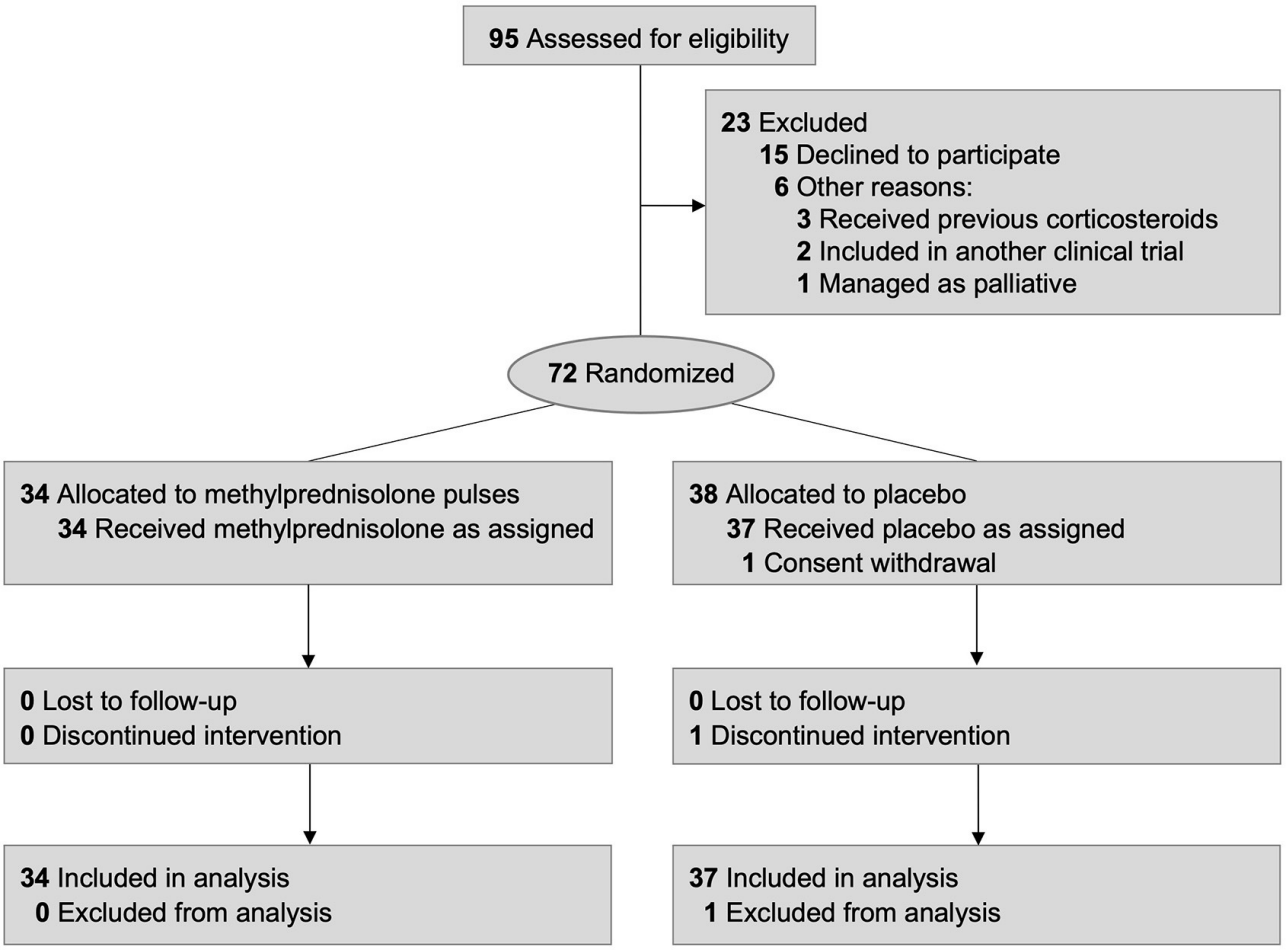
Participant flow diagram.

A total of 3,453 patients with confirmed SARS-CoV-2 pneumonia were admitted to the two enrolling hospitals during the recruitment period. Participants were enrolled between 8 May 2020 and 13 March 2021. Candidate patients were identified by the investigators and collaborators, but not all admitted COVID-19 patients were screened. The majority of patients were directly excluded by study collaborators sited at the Emergency Department because of the existence of respiratory failure, need for supplementary oxygen or dexamethasone administration at Emergency Department. Thus, we identified 95 patients who met the inclusion criteria and none of the exclusion criteria. Of these, 72 were randomized to receive either MPP (34 patients) or placebo (38 patients). One patient of the placebo group withdrew from the study before receiving the first dose of the allocated intervention and was excluded from the modified intention-to-treat analysis. Another patient of the placebo group discontinued the intervention due to receiving unmasked MP before the criteria for treatment failure were met, but the case was included in the modified intention-to-treat analysis (Figure 1). The follow-up period ended on 9 April 2021, once achieved the established sample size.

### Baseline Characteristics and Adjuvant Therapies

No significant differences in baseline characteristics were identified between both groups (Table 1). Overall, the median age was 58.4 years (48.1–69.0), and there was male predominance (69%). The Caucasian race was the most frequent (60.6%), with Hispanic ethnicity accounting for 33.8% of the patients. The most frequent comorbidities were arterial hypertension (32.4%) and diabetes (18.3%). The median time elapsed from symptom onset was nine (8–10) days. The median baseline PaO_2_ and SpO_2_ were 73.1 mmHg (68.2–80.2) and 95% (95–96%), respectively. At baseline, the CRP level was >60 mg/L in 58 (81.7%) patients; in the remaining thirteen patients, the median CRP was 42.7 (20.3–45.8) mg/L and the inclusion laboratory criterion was based on ferritin level, with a median of 1338.0 (1104.0–1739.0) μg/L.

**Table 1:** Baseline patient characteristics.

	**Total (*N* = 71)**	**MPP group (*N* = 34)**	**Control group (*N* = 37)**	***p***
Age, *years*	58.4 (48.1–69.0)	61.1 (49.9–66.2)	57.4 (45.8–69.4)	0.85
Sex, *male (%)*	49 (69)	26 (76.5)	23 (62.2)	0.19
**Race/ethnicity**, ***n (%)***				
Caucasian	43 (60.6)	21 (61.8)	22 (59.5)	
Hispanic	24 (33.8)	10 (29.4)	14 (37.8)	0.40
Other	4 (5.6)	3 (8.8)	1 (2.7)	
Body mass index	28.7 (26.4–30.5)	28.9 (27.0–30.3)	27.6 (24.5–31.2)	0.24
**Smoking status**, ***n (%)***				
Never smoker	45 (63.4)	21 (61.8)	24 (53.3)	0.78
Former smoker	24 (33.8)	13 (38.2)	11 (29.7)	0.45
Current smoker	2 (2.8)	0 (0)	2 (5.44)	0.49
Arterial hypertension, *n (%)*	23 (32.4)	11 (32.4)	12 (32.4)	0.99
Diabetes mellitus, *n (%)*	13 (18.3)	8 (23.5)	5 (13.5)	0.27
Renal failure, *n (%)*	5 (7)	3 (8.8)	2 (5.4)	0.66
Heart failure, *n (%)*	0 (0)	0 (0)	0 (0)	
**Respiratory disease**, ***n (%)***	6 (8.5)	2 (5.9)	4 (10.8)	0.67
COPD	1 (1.4)	1 (2.9)	0 (0)	
Asthma	2 (2.8)	0 (0)	2 (5.4)	0.35
Other^**#**^	3 (4.2)	1 (2.9)	2 (5.4)	
Previous cancer, *n (%)*	3 (4.2)	2 (5.9)	1 (2.7)	0.60
Charlson index	0 (0–1)	0 (0–1)	0 (0–1)	0.44
**Ongoing pharmacological treatment**, ***n (%)***				
Oral antidiabetics	11 (15.5)	6 (17.6)	5 (13.5)	0.63
Insulin	5 (7.0)	3 (8.8)	2 (5.4)	0.66
ACEI or ARB	16 (22.5)	7 (20.6)	9 (24.3)	0.71
Inhaled corticosteroids	2 (2.8)	0 (0)	2 (5.4)	0.49
Hydroxychloroquine	0 (0)	0 (0)	0 (0)	
Other	37 (52.1)	18 (52.9)	19 (51.4)	0.89
Time from symptom onset, *days*	9 (8–10)	9 (7–10)	9 (8–11)	0.20
Oxygen saturation, %	95 (95–96)	95 (95–96)	96 (94.5–96.5)	0.51
SpO_2_/FiO_2_	452 (447–457)	452 (447–457)	452 (448–457)	0.81
PaO_2_, *mmHg*	73.1 (68.2–80.2)	73.0 (70.8–86.2)	73.2 (67.0–77.1)	0.32
**Chest X-ray**, ***n (%)***				
Lobar pneumonia	13 (18.3)	7 (20.6)	6 (16.2)	
Bilobar pneumonia	27 (38.0)	12 (35.3)	15 (40.5)	0.96
Multilobar pneumonia	29 (40.8)	14 (41.2)	15 (40.5)	
Consolidations	2 (2.8)	1 (2.9)	1 (2.7)	
**Laboratory parameters**				
Lymphocyte count, *10^9^/L*	1,150 (900–1,530)	1,110 (800–1,625)	1,200 (980–1,450)	0.88
Platelet count, *10^12^/L*	211 (162–300)	205.5 (158–288)	223 (169–310)	0.30
C-reactive protein, *mg/L*	93.0 (66.5–126.0)	92.7 (65.0–122.9)	95.2 (68.9–130.6)	0.83
Ferritin, *μg/L*	743.0 (393.2–1172.9)	737.0 (383.1–1169.0)	754.0 (398.5–1173.9)	0.83
Interleukin-6, *pg/ml*	30.7 (17.4–48.2)	34.8 (18.1–48.7)	28.7 (15.8–47.0)	0.53
LDH, IU/L	296.0 (253.0–358.2)	294.5 (260.0–373.2)	301.0 (251.5–355.2)	0.89
D-dimer, *ng/ml*	563.5 (400.0–812.0)	630.0 (414.5–954.0)	534.0 (391.0–723.0)	0.45

*Quantitative data are expressed as median (interquartile range) and categorical data as number of patients (%)*.

*MPP, methylprednisolone pulses; COPD, chronic obstructive pulmonary disease; ACEI, angiotensin-converting enzyme inhibitor; ARB, angiotensin-receptor blocker; SpO_2_/FiO_2_, ratio between the peripheral oxygen saturation (SpO_2_) and the fraction of inspired oxygen (FiO_2_); PaO_2_, arterial partial pressure of oxygen; LDH, lactate dehydrogenase*.

# *Including two cases of bronchiectasis and one case of obstructive sleep apnea syndrome*.

Remdesivir was used in six (8.5%) patients, two (5.9%) in the MPP group and four (10.8%) in the control group (*p* = 0.67). After achieving the primary outcome, 20 (28.2%) patients were treated with tocilizumab and unmasked corticosteroids; of them, 13 (18.3%) patients received MP and seven (9.9%) received dexamethasone as per treating physician's discretion.

### Primary Outcome

Overall, 20 (28.2%) patients presented with treatment failure: ten in each group (29.4% in the MPP group vs. 27% in the control group, *p* = 0.82). The components of the primary outcome are detailed in Table 2. Of the 20 patients who developed treatment failure, all met one of the two conditions for clinical worsening. In 17 of these 20 patients (85%), the clinical worsening was due to a fall of SpO_2_ in ambient air below 90%, PaO_2_ in ambient air below 60 mmHg, or PaO_2_/FiO_2_ ratio below 300 mmHg (condition #1 of *clinical worsening*). There were no differences in the proportion of treatment failures defined by condition #1 of *clinical worsening* between MPP and control groups (9/10 vs. 8/10, respectively; *p* = 0.63). There were no cases of death or MV requirements within 14 days of the randomization. Only one patient of the placebo group was admitted to the ICU on day 14 post-randomization and received high-flow oxygen therapy. The time to treatment failure was similar between the MPP and control groups (1 [1.0–3.2] vs. 1.5 [0.7–2.0] days, respectively; *p* = 0.74 [Figure 2]).

**Table 2:** Main outcomes.

	**Total (*N* = 71)**	**MPP group (*N* = 34)**	**Control group (*N* = 37)**	***p***
**Primary outcome**				
Treatment failure at 14 days, *n (%)*	20 (28.2)	10 (29.4)	10 (27)	0.823
Mortality	0 (0)	0 (0)	0 (0)	
ICU admission	1 (1.4)	0 (0)	1 (2.7)	1.000
Need for mechanical ventilation	0 (0)	0 (0)	0 (0)	
Clinical worsening^**#**^	20 (28.2)	10 (29.4)	10 (27)	0.823
**Secondary outcomes**				
Mortality at 28 days, *n (%)*	0 (0)	0 (0)	0 (0)	
ICU admission at 28 days, *n (%)*	1 (1.4)	0 (0)	1 (2.7)	1.000
Need for high-flow oxygen therapy, *n (%)*	4 (5.6)	1 (2.9)	3 (7.9)	0.360
Radiological worsening, *n (%)*	32 (45.1)	13 (38.2)	19 (51.4)	0.267
Length of hospital stay, *days*	7 (5–10)	6 (5–10)	7 (5–11)	0.237

*Data are expressed as number of patients (%) and median (interquartile range)*.

*MPP, methylprednisolone pulses; ICU, intensive care unit*.

# *Defined as at least one of the two following conditions: (1) SpO_2_ in ambient air below 90%, or PaO_2_ in ambient air below 60 mmHg or PaO_2_/FiO_2_ below 300; or (2) ≥15% decrease in the PaO_2_ from baseline, together with an increase in the levels of any inflammatory marker (C-reactive protein, interleukin-6, or ferritin) or radiological progression*.

**Figure 2:**
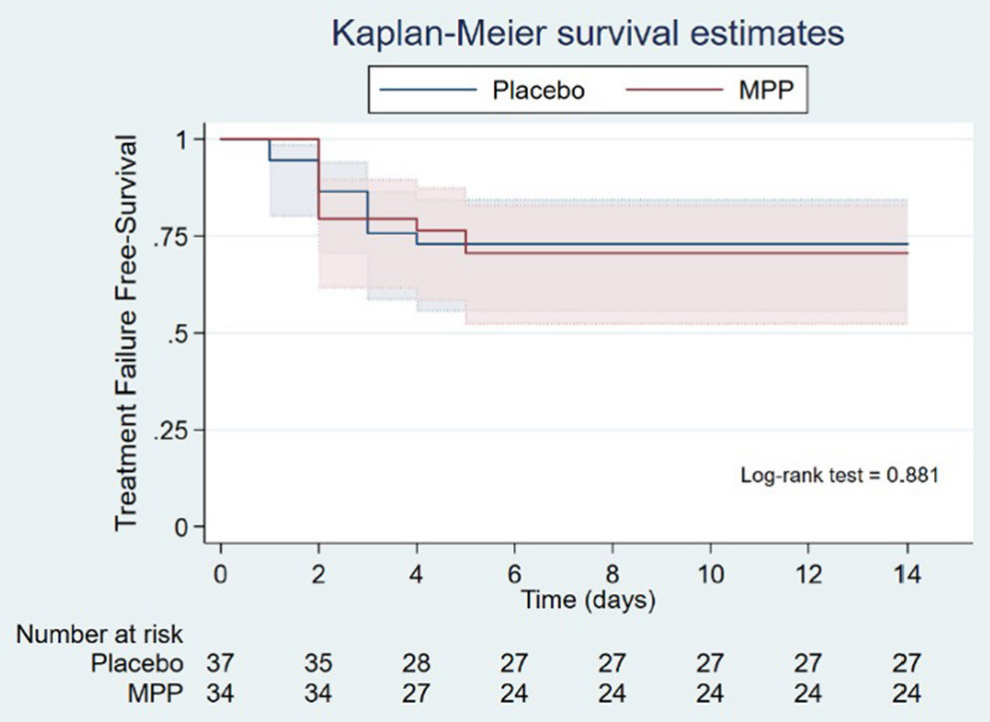
Kaplan-Meier survival curves of the time to primary outcome (treatment failure) in the MPP and placebo groups. The time to primary outcome of the study (treatment failure) was analyzed with the Kaplan-Meier estimator and compared between the MPP and control groups using the log-rank Mantel-Cox test (1.0 [1.0–3.2] days in the MPP group vs. 1.5 [0.7– 2.0] days in the placebo group, *p* = 0.74). MPP, methylprednisolone pulses.

### Secondary Outcomes

The secondary outcomes are detailed in Table 2. No deaths occurred by 28 days post-randomization, and only one patient of the control group required ICU admission by day 28. There were no significant differences between the MPP and control groups regarding the need for high-flow oxygen therapy, radiological worsening, or the length of hospitalization.

**Figure 3:**
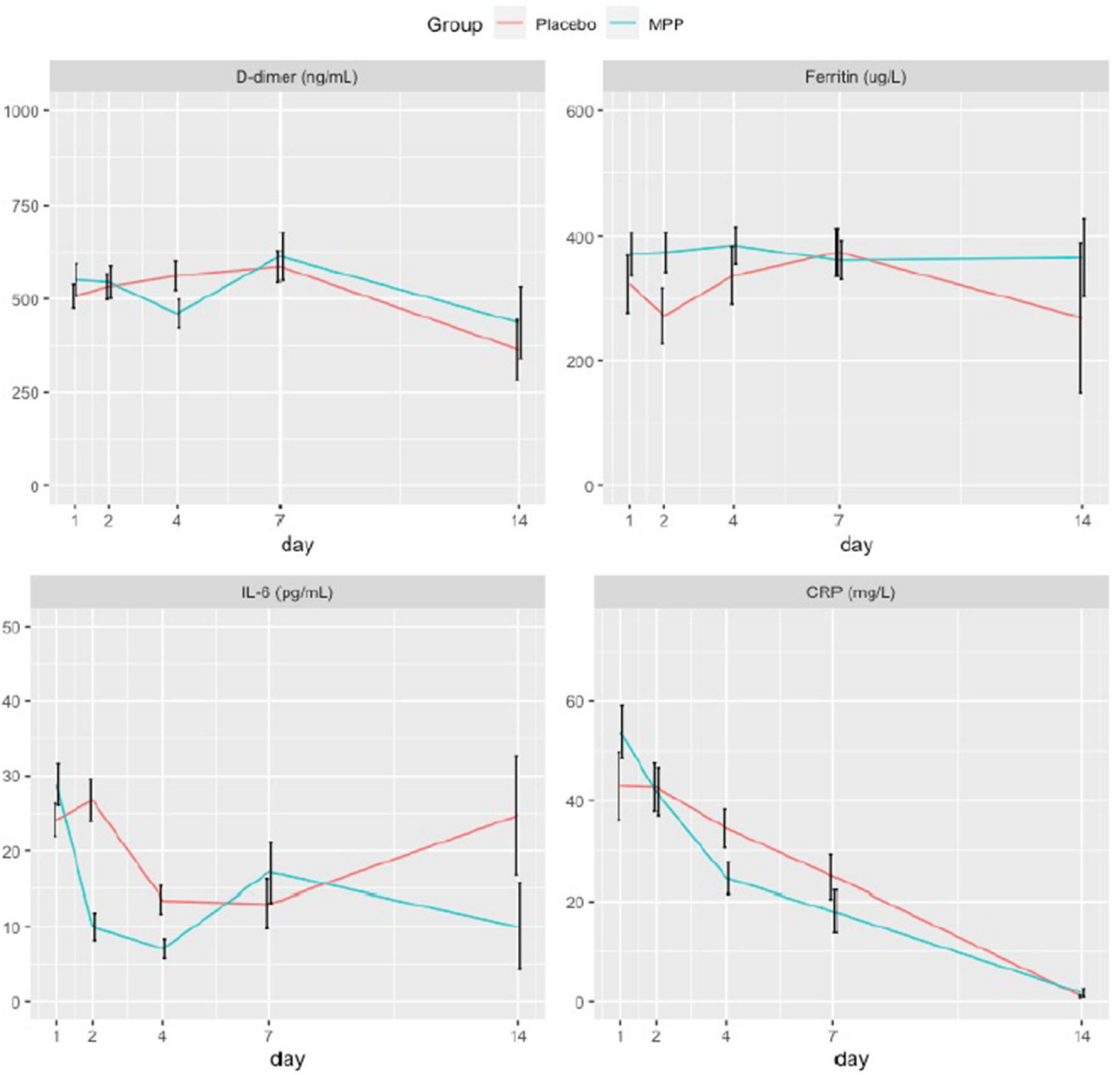
Temporal evolution of the study participants' inflammatory markers. These plots show the changes in the serial measurements of inflammatory laboratory parameters performed over a period of 14 days. Colored lines connect the mean value and black vertical lines indicate the confidence intervals of each measurement. Ten patients of the MPP group and another ten of the placebo group were treated with tocilizumab, a monoclonal antibody that blocks the IL-6 receptor and may cause a reactive increase in IL-6 plasma levels, within a median time of one day. MPP, methylprednisolone pulses; IL-6, interleukin-6; CRP, C-reactive protein.

Concerning the laboratory parameters (Figure 3), the only significant difference was a higher decrease in the levels of CRP at day 4 post-randomization in the MPP group (Supplementary Tables S1–S3).

### Adverse Events

Overall, 36 adverse events were recorded in 32 (41.5%) patients (Table 3), without significant differences between the MPP and control groups (50.0 vs. 40.5%, *p* = 0.42). All adverse events were considered to be non-serious by the attending physicians and did not lead to a delay in the participants' hospital discharge or readmission. Decompensation of previous diabetes mellitus occurred more frequently in the MPP group (26.5 vs. 5.4%, *p* = 0.02). Infections other than COVID-19 occurred more commonly in the control group (16.2 vs. 0.0%, *p* = 0.02).

**Table 3:** Adverse events at day 28.

	**Total (*N* = 71)**	**MPP group (*N* = 34)**	**Control group (*N* = 37)**	***p***
Number of AEs	36	18	18	0.776
Patients with at least one AE, *n (%)*	32 (41.5)	17 (50)	15 (40.5)	0.424
Decompensation of diabetes mellitus, *n (%)*	11 (15.5)	9 (26.5)	2 (5.4)	0.021
Decompensation of arterial hypertension, *n (%)*	0 (0)	0 (0)	0 (0)	
Infection other than COVID-19^**#**^, *n (%)*	6 (8.6)	0 (0)	6 (16.2)	0.026
Psychosis or mania, *n (%)*	0 (0)	0 (0)	0 (0)	
Dyspepsia, *n (%)*	2 (2.8)	0 (0)	2 (5.4)	0.494
Gastrointestinal bleeding, *n (%)*	0 (0)	0 (0)	0 (0)	
Arterial thrombosis, *n (%)*	0 (0)	0 (0)	0 (0)	
Venous thrombosis, *n (%)*	1 (1.4)	1 (2.9)	0 (0)	0.479
Other, *n (%)*	16 (22.5)	8 (23.5)	8 (21.6)	0.848
SARS-CoV-2 positivity at 7 days as determined by RT-PCR testing^¶^, *n (%)*	47/66 (66.2)	23/32 (71.9)	24/34 (70.6)	0.908

*Data are expressed as number of patients (%)*.

*MPP, methylprednisolone pulses; AE, adverse event; RT-PCR, reverse-transcription polymerase chain reaction*.

# *Including three cases of catheter-related phlebitis, two of mucosal candidiasis, and one of bacterial pneumonia*.

¶ *Defined as a cycle threshold ≤ 35 in a RT-PCR performed on a nasopharyngeal swab sample*.

Forty-seven out of 66 patients (66.2%) did not achieve SARS-CoV-2 clearance in a nasopharyngeal RT-PCR test performed seven days post-randomization, but no significant differences were observed between the MPP and control groups (71.9 vs. 70.6%, *p* = 0.91).

### Factors Associated With Treatment Failure

Data of the bivariate and multivariate analyses are summarized in Table 4 and detailed in the Supplementary Table S4. In the multivariate analysis, three baseline factors were identified as independent predictors of treatment failure: platelet count (OR 1.00, 95% confidence interval [CI] 1.00–1.00, *p* = 0.012), IL-6 (OR 1.02, 95% CI 1.00–1.04, *p* = 0.030), and SpO_2_/FiO_2_ values (OR 0.97, 95% CI 0.94–0.99, *p* = 0.014).

**Table 4:** Logistic regression of baseline variables associated with treatment failure.

	**Bivariate analysis**			**Multivariate Analysis**		
	**OR**	**95% CI**	***p***	**OR**	**95% CI**	***p***
Lymphocyte count	0.99	0.99–1.00	0.04	1.00	0.99–1.00	0.39
Platelet count	1.00	1.00–1.00	0.003	1.00	1.00–1.00	0.01
Interleukin-6	1.01	1.00–1.03	0.03	1.02	1.00–1.04	0.03
SpO_2_/FiO_2_	0.98	0.96–0.99	0.04	0.97	0.94–0.99	0.01
Time from symptom onset	0.60	0.42–0.86	0.005	0.78	0.57–1.06	0.12

*A bivariate analysis was first performed with a logistic regression and a multivariate analysis was subsequently performed with a logistic regression applying a backward stepwise process. Variables with a P value below 0.05 were included in the multivariate analysis*.

*OR, odds ratio; CI, confidence interval; SpO_2_/FiO_2_, ratio between the peripheral oxygen saturation (SpO_2_) and the fraction of inspired oxygen (FiO_2_)*.

## Discussion

The main finding of the CORTIVID trial is that a 3-day course of MPP administered after the first week of symptom onset did not prevent progression to respiratory failure nor death in hospitalized COVID-19 patients with raised inflammatory markers who did not require oxygen at baseline.

Despite the initial concerns on their potentially harmful effects in COVID-19 patients (15), corticosteroids were used early in the pandemic due to their beneficial effect in ARDS (16) and the recognized inflammatory pathophysiology of the COVID-19 (7). In a quasi-experimental study, Fadel et al. described a decrease in mortality, ICU admission, and MV rates among patients requiring oxygen therapy who were treated with MP (17). However, the paradigm shift in favor of corticosteroids occurred with the publication of the preliminary results of the RECOVERY trial, which demonstrate a 28-day survival benefit from dexamethasone (6 mg/day) in hospitalized COVID-19 patients requiring respiratory support (11). Afterwards, an open-label RCT carried out by Corral-Gudino et al. confirmed that MP at an initial dose of 40 mg twice daily was associated with better outcomes in patients with respiratory failure (18). A subsequent single-blind RCT performed by Edalatifard et al. also described a decrease in mortality with the use of a 3-day course of pulsed MP in non-critically ill patients with respiratory failure (19). However, other RCTs were unable to demonstrate such benefit arising from treatment with corticosteroids (20–23). In the first double-blind trial conducted with corticosteroids, Jeronimo et al. found no survival benefit from MP, although this could be explained by the long time elapsed from disease onset to randomization (20). In a multicenter, open-label RCT, Tomazini et al. observed no benefits on survival rates after a 10-day course of dexamethasone in COVID-19 patients with severe ARDS. However, it should be noted that 35% of the patients in the control group received corticosteroids (21). Finally, neither the REMAP-CAP COVID-19 nor the CAPE COVID-19 trials demonstrated a decrease in mortality with the use of hydrocortisone in critically ill COVID-19 patients (22, 23). Differences between the corticosteroids used in these trials with respect to their affinity for the glucocorticoid receptor, intrinsic anti-inflammatory effect, and mineralocorticoid potency could possibly explain the negative results obtained with hydrocortisone and the positive outcomes achieved with dexamethasone and MP (9, 24, 25). Overall, the beneficial effect of corticosteroids on short-term mortality and need for MV was confirmed in two meta-analyses (26, 27).

In almost all these studies, treatment with corticosteroids was limited to patients who had developed respiratory failure. However, in clinical practice, there are patients who are admitted for SARS-CoV-2 pneumonia beyond the first week of symptoms with increased levels of inflammatory markers but not respiratory failure. We hypothesized that early corticosteroid treatment could halt the ongoing inflammatory response in these patients and prevent respiratory deterioration. We used a 3-day course of MPP based on the rationale that the non-genomic pathway activated by pulses produces a quicker anti-inflammatory effect while minimizing potential adverse events owing to the short treatment duration (28). However, we observe neither a decrease in the rate of progression to respiratory failure nor a survival benefit with MPP. It is possible that COVID-19 patients with increased levels of inflammatory markers but not respiratory failure after 7 days since symptom onset may already represent a subgroup of patients with good prognosis. It should be noted that most patients (85.9%) included in this study had a baseline SpO_2_ ≥94% in ambient air. The use of MPP may have yielded positive results in patients with a baseline SpO_2_ of 90–93%, as seven of the 20 primary outcomes were achieved in the subgroup of ten patients who presented with an ambient air SpO_2_ < 94% at inclusion. Altogether, our findings support the results obtained in the RECOVERY trial, highlighting that COVID-19 patients without oxygen requirements do not benefit from corticosteroids. Future RCTs should clarify whether high-dose corticosteroid therapy is more effective than dexamethasone at the currently established dosage and, specifically, if pulse-based regimens are superior to non-pulse-based ones (29).

Our study has several limitations that must be recognized. First, the sample size was small and its calculation was somehow tentative, and the trial was underpowered to detect a difference in treatment failure of 25% between the MPP and control groups, as we initially expected. It should be noted that at the moment of the trial design (March and April 2020), little information was available beyond clinical observations on the impact of MPP on COVID-19. Second, a 3-day course of MPP may be excessively short considering the natural history of the disease. Third, in the context of the pandemic waves lived in Spain and shortcomings in the trial organization due to the lack of financial support, we were unable to perform an individual screening of most patients who arrived at the Emergency Department. However, the criterion applied to exclude the vast majority of patients was the existence of respiratory failure so that, on a practical basis, patients who required supplementary oxygen were directly excluded without being screened. Fourth, the restrictive inclusion criteria resulted in a highly selected eligible population; therefore, our results are generalizable only to the particular subgroup of patients without oxygen requirements but with increased levels of inflammatory markers at admission. Even so, it is striking that no patient in our series died during the follow-up, especially those older than 75 years. This low mortality reinforces the idea that our population was highly selected. Another explanation could be that patients included in the trial were closely monitored and rescued very early once they met the criteria for treatment failure. Lastly, the findings of our study may not be applicable to COVID-19 caused by delta or omicron variants.

In conclusion, a 3-day course of MPP administered after the first week of symptom onset did not prevent clinical deterioration in hospitalized COVID-19 patients with increased levels of inflammatory markers but no oxygen requirements.

## Data Availability Statement

The original contributions presented in the study are included in the article/Supplementary Material, further inquiries can be directed to the corresponding author/s.

## Ethics Statement

The studies involving human participants were reviewed and approved by Comité Ético de Investigación Clínica con Medicamentos (CEIm) del Hospital Universitario de la Princesa (Madrid). The patients/participants provided their written informed consent to participate in this study.

## Author Contributions

All authors contributed to the production of this manuscript and have approved the final version.

## Conflict of Interest

The authors declare that the research was conducted in the absence of any commercial or financial relationships that could be construed as a potential conflict of interest.

## Publisher's Note

All claims expressed in this article are solely those of the authors and do not necessarily represent those of their affiliated organizations, or those of the publisher, the editors and the reviewers. Any product that may be evaluated in this article, or claim that may be made by its manufacturer, is not guaranteed or endorsed by the publisher.
