# Supplementary material for: Methylprednisolone Pulses in Hospitalized COVID-19 Patients Without Respiratory Failure: A Randomized Controlled Trial
Source: Front Med (Lausanne). 2022 Feb 28;9:807981. doi: 10.3389/fmed.2022.807981 (PMC8919087; doi:10.3389/fmed.2022.807981)
Supplement: Supplementary file 1 [file Data_Sheet_1.docx]

Supplementary Material

**Supplementary Table S1. Changes in the Laboratory Parameters at Day 4 Post-Randomization**

|  | **Overall**  (*N* = 71) | **MPP group**  (*N* = 34) | **Control group**  (*N* = 37) | ***p*** |
| --- | --- | --- | --- | --- |
| Lymphocyte count, *10^9^/L* | +254.28 ± 542.06 | +272.05 ± 668.75 | +237.50 ± 396.24 | 0.79 |
| Platelet count, *10^12^/L* | +912.85 ± 662.74 | +959.39 ± 578.26 | +871.35 ± 735.43 | 0.58 |
| C-reactive protein, *mg/L* | -45.95 ± 57.99 | **-69.86 ± 51.04** | **-24.69 ± 56.05** | **0.001** |
| Ferritin, *µg/L* | -53.10 (-229.00-143.50) | -56.80 (-231.00-82.02) | +36.60 (-226.05-195.00) | 0.69 |
| IL-6, *pg/ml* | -8.00 (-29.75-23.20) | -13.10 (-34.15-6.55) | -5.60 (-19.22-47.15) | 0.17 |
| LDH, *U/L* | -36.00 (-82.90-4.50) | -32.00 (-84.40-6.00) | -39.60 (82.80-2.25) | 0.95 |
| D-dimer, *ng/ml* | -99.00 (-238.00-107.50) | -119.00 (-291.50-92.50) | -32.50 (-221.50-108.75) | 0.60 |

Quantitative variables were expressed as mean ± standard deviation or median (interquartile range) and compared between groups using the Student's t-test for unpaired data or the Mann-Whitney U test depending on whether the data were normally distributed according to the Kolmogorov-Smirnov test.

IL-6: interleukin-6; LDH: lactate dehydrogenase.

**Supplementary Table S2. Changes in the Laboratory Parameters at Day 7 Post-Randomization**

|  | **Overall**  (*N* = 49) | **MPP group**  (*N* = 20) | **Control group**  (*N* = 29) | ***p*** |
| --- | --- | --- | --- | --- |
| Lymphocyte count, *10^9^/L* | +659.18 ± 675.16 | +786.00 ± 939.57 | +571.72 ± 403.75 | 0.34 |
| Platelet count, *10^12^/L* | +164.83 ± 100.45 | +168.05 ± 721.85 | +162.26 ± 117.25 | 0.84 |
| C-reactive protein, *mg/L* | -75.74 ± 69.27 | -89.63 ± 91.16 | -65.81 ± 47.53 | 0.24 |
| Ferritin, *µg/L* | -85.00 (-323.00-68.77) | -292.39 (-400.50-84.75) | -58.00 (-320.30-23.25) | 0.69 |
| IL-6, *pg/ml* | -14.80 (-34.62-19.35) | -17.30 (-49.00-0.00) | -12.50 (-33.00-34.75) | 0.17 |
| LDH, *U/L* | -58.07 ± 82.94 | -58.50 ± 64.16 | -57.77 ± 94.86 | 0.98 |
| D-dimer, *ng/ml* | -20.00 (-217.50-349.50) | +17.00 (-206.25-376.50) | -24.00 (-230.00-264.50) | 0.60 |

Quantitative variables were expressed as mean ± standard deviation or median (interquartile range) and compared between groups using the Student's t-test for unpaired data or the Mann-Whitney U test depending on whether the data were normally distributed according to the Kolmogorov-Smirnov test.

IL-6: interleukin-6; LDH: lactate dehydrogenase.

**Supplementary Table S3. Changes in the Laboratory Parameters at Day 14 Post-Randomization**

|  | **Overall**  (*N* = 12) | **MPP group**  (*N* = 6) | **Control group**  (*N* = 6) | ***p*** |
| --- | --- | --- | --- | --- |
| Lymphocyte count, *10^9^/L* | +1005.00 ± 634.87 | +773.33 ± 538.57 | +1236.66 ± 684.07 | 0.22 |
| Platelet count, *10^12^/L* | +50.23 ± 123.23 | 33.42 ± 170.22 | 69.83 ± 61.93 | 0.63 |
| C-reactive protein, *mg/L* | -100.90 ± 117.72 | -151.54 ± 130.43 | -50.26 ± 85.55 | 0.14 |
| Ferritin, *µg/L* | -565.34 ± 1019.14 | -808.88 ± 1484.08 | -356.60 ± 362.97 | 0.45 |
| IL-6, *pg/ml* | -54.48 ± 95.40 | -74.02 ± 129.27 | -34.95 ± 59.21 | 0.60 |
| LDH, *U/L* | -71.80 ± 97.67 | -124.70 ± 60.88 | -8.32 ± 99.91 | 0.12* |
| D-dimer, *ng/ml* | -250.00 ± 429.12 | -402.33 ± 483.81 | -67.20 ± 301.99 | 0.21 |

Quantitative variables were expressed as mean ± standard deviation or median (interquartile range) and compared between groups using the Student's t-test for unpaired data or the Mann-Whitney U test depending on whether the data were normally distributed according to the Kolmogorov-Smirnov test.

*Performed with the Mann-Whitney U test due to the low number of patients analyzed (*n* = 12).

**Supplementary Table S4. Bivariate Analysis of Variables Associated with Occurrence of the Primary Outcome (Treatment Failure)**

| **Factor** | **Treatment failure**  (*N* = 20) | **No treatment failure**  (*N* = 51) | ***p*** |
| --- | --- | --- | --- |
| ***Demographic features*** |  |  |  |
| Age, *years* | 60.93 ± 12.56 | 57.16 ± 13.94 | 0.29 |
| Male sex | 14/49 (28.6) | 35 /49 (71.4) | 0.91 |
| Race  Hispanic  Non-Hispanic | 5/24 (20.8)  15/47 (31.9) | 19/24 (79.2)  32/47 (68.1) | 0.32 |
| Smoking status  Smoker (prior or current)  Never smoker | 33/51 (64.7)  12/20 (60) | 18/51 (35.3)  8/20 (40) | 0.71 |
| Body mass index, *kg/m^2^* | 28.70 ± 4.49 | 28.69 ± 4.72 | 0.99 |
| ***Comorbidities*** |  |  |  |
| Charlson index | 0 (0-1) | 0 (0-1) | 0.41 |
| Arterial hypertension | 7/23 (30.4) | 16/23 (69.6) | 0.77 |
| Diabetes mellitus | 2/13 (15.4) | 11/13 (84.6) | 0.32 |
| Prior respiratory disease | 1/6 (16.7) | 5/6 (83.3) | 0.67 |
| ***Previous chronic pharmacological treatment*** |  |  |  |
| Oral antidiabetics | 2/11 (18.2) | 9/11 (81.8) | 0.72 |
| Insulin | 0/5 (0) | 5/5 (100) | 0.14 |
| ACEI or ARB | 3/16 (18.8) | 13/16 (81.3) | 0.53 |
| Inhaled corticosteroid | 1/2 (50) | 1/2 (50) | 0.48 |
| ***Clinical presentation*** |  |  |  |
| Time from symptom onset, *days* | 8 (6.25-9) | 10 (8-11) | **0.001** |
| SpO_2_/FiO_2_ at admission | 450 (442.8-457.1) | 452.3 (452.3-457.1) | **0.07** |
| ***Radiological features*** |  |  |  |
| Multilobar pneumonia | 9/31 (29) | 22/31 (71) | 0.89 |
| Non-multilobar pneumonia | 11/40 (27.5) | 29/40 (72.5) |  |
| ***Laboratory parameters*** |  |  |  |
| Hemoglobin, *g/dl* | 13.94 ± 1.68 | 13.86 ± 1.49 | 0.86 |
| Lymphocyte count, *10^9^/L* | 1027.50 ± 470.76 | 1311.37 ± 513.99 | **0.03** |
| Platelet count, *10^9^/L* | 174600 ± 78264 | 251921 ± 94711 | **0.002** |
| LDH, *IU/L* | 311 (277.4-370.2) | 296 (247-352.2) | 0.31 |
| D-dimer, *ng/ml* | 665.5 (389-818) | 533.5 (420.2-828.5) | 0.76 |
| Fibrinogen | 678.63 ± 133.55 | 734.16 ± 147.31 | 0.19 |
| Interleukin-6, *pg/ml* | 40 (22.5-80) | 28.7 (15.8-44) | **0.07** |
| C-reactive protein, *mg/L* | 101.94 ± 85.74 | 98.61 ± 41.93 | 0.83 |
| Ferritin, *µg/L* | 1052.5 (431.2-1386.7) | 728.4 (366.8-1115.2) | 0.12 |
| Procalcitonin, *ng/ml* | 0.06 (0.05-0.11) | 0.06 (0.04-0.11) | 0.53 |
| ***In-hospital treatments*** |  |  |  |
| Hydroxychloroquine | 1/5 (20) | 4/5 (80) | 0.67 |
| Lopinavir/ritonavir | 3/4 (75) | 1/4 (25) | 0.06 |
| Remdesivir | 3/3 (50) | 3/3 (50) | 0.34 |

Categorical variables were expressed as frequencies (percentages) and compared between patients with and without treatment failure using Pearson's *χ*^2^ and Fisher's exact tests depending on the expected cell frequencies.

Quantitative variables were expressed as mean ± standard deviation or median (interquartile range) and compared between patients with and without treatment failure using the Student's *t*-test for unpaired data or the Mann-Whitney *U* test depending on whether the data were normally distributed according to Kolmogorov‑Smirnov test.

ACEI: angiotensin-converting enzyme inhibitor; ARB: angiotensin-receptor blocker.
